# Evaluation of clinical, analytical, and genotyping performance of Hex L1 real-time PCR coupled with high-resolution melting curve analysis for fowl adenovirus outbreak investigation in Morocco

**DOI:** 10.3389/fvets.2025.1654833

**Published:** 2025-11-26

**Authors:** Amina Kardoudi, Abdelouaheb Benani, Faouzi Kichou, IKram Ouchhour, Khaoula Erafii, Siham Fellahi

**Affiliations:** 1 Department of Veterinary Pathology and Public Health, Hassan 2nd Institute of Agronomy and Veterinary Medicine, Rabat, Morocco; 2 Medical Biology Department, Molecular Biology Laboratory, Pasteur Institute of Morocco, Casablanca, Morocco; 3 African BioGenome Center, Mohammed VI Polytechnic University, Ben Guerir, Morocco

**Keywords:** outbreaks, IBH, age, FAdV, real-time PCR, genotyping, HRM

## Abstract

Fowl adenoviruses (FAdVs) are widespread viruses in poultry populations, responsible for several severe diseases, including Inclusion Body Hepatitis (IBH), Adenoviral Gizzard Erosion (AGE), and Hepatitis-Hydropericardium Syndrome (HHP). These diseases have been associated with significant economic and health impacts on poultry industries. Accurate detection and genotyping play a key role in the diagnosis of these infections, as different FAdV genotypes are associated with distinct disease syndromes and epidemiological patterns. In this study, we aimed to evaluate the clinical, analytical, and genotyping performance of the Hex L1 PCR combined with High-Resolution Melting (HRM) Curve analysis for investigating recent IBH and AGE outbreaks in Morocco. The study involved 26 clinical samples collected from broiler and layer poultry farms suspected with IBH or AGE. These samples were amplified using conventional PCR, real-time PCR/52 K test, and the Hex L1 PCR/HRM test. Field samples were also sequenced and compared with HRM curve analysis results to validate the genotyping accuracy of the Hex L1 PCR/HRM method. Phylogenetic analysis of the sequenced samples revealed several FAdV genotypes, including FAdV-11 and FAdV-8b in IBH cases, and FAdV-1 and FAdV-8a in AGE cases, highlighting the genetic diversity of circulating strains. The Hex L1 PCR/HRM method successfully amplified all 12 FAdV serotypes, demonstrating excellent reproducibility and repeatability, with coefficients of variation ranging from 0.19% to 1.82%. Moreover, this method showed a strong correlation with the real-time PCR/52 K test, achieving a high correlation coefficient of 0.9077. The HRM curve analysis accurately genotyped all the field samples, with results consistent with sequencing outcomes. In conclusion, this method provides a fast, sensitive, and reliable alternative for FAdV detection and genotyping. It enables universal detection, quantification, and genotyping in a single step, overcoming the limitations of traditional techniques, making it an ideal tool for sample screening, while sequencing validation is necessary for confirmation.

## 1 Introduction

Fowl adenoviruses (FAdVs) belong to the *Adenoviridae* family and the *Aviadenovirus* genus, encompassing avian adenoviruses that share common antigens. To date, five FAdV species (FAdV-A to FAdV-E) have been identified by complete genome analysis using restriction endonucleases, while viral neutralization (VN) test has identified 12 serotypes (1–7, 8a, 8b, 9–11) (1, 2). These serotypes are distributed among the different species: FAdV-A includes serotype 1; FAdV-B includes serotype 5; FAdV-C comprises serotypes 4 and 10; FAdV-D comprises serotypes 2, 3, 9, and 11; and FAdV-E comprises serotypes 6, 7, 8a, and 8b. FAdVs possess a linear double-stranded DNA genome, with a size that varies slightly among the 12 serotypes (ranging from ~43 to 45 kb) (3).

These viruses are associated with diseases such as inclusion body hepatitis (IBH) (4, 5), adenoviral gizzard erosion (AGE) (6, 7), pancreatic necrosis (8), and hepatitis-hydropericardium syndrome (HHS) (7). Accurate detection and identification of the strains responsible for these outbreaks is essential for epidemiological tracing and for the implementation of effective control strategies, including vaccination.

Viral isolation followed by electron microscopy has traditionally been used for diagnosing FAdV infections (1). However, this approach is time-consuming and requires fresh samples, although it still remains the gold standard reference technique for confirming FAdV infections. In addition, various serological tests have been widely employed for FAdV detection, including agar gel immunodiffusion (9), immunofluorescence (10), counter immunoelectrophoresis (11), and agar gel precipitation tests (12). Among these, VN test is considered the gold standard for differentiating between FAdV serotypes (1). Despite its accuracy, the VN test has significant drawbacks that limit its use for mass detection, such as high cost, the need for cell culture and reference strains, and the considerable time required. Moreover, cross-reactivity between serotypes within the same species complicates interpretation. For instance, FAdV-11 can cross-react with FAdV-2 (13), and FAdV-4 with FAdV-10 (14), leading to challenges in obtaining definitive results.

Compared with other techniques, molecular techniques offer significant advantages in terms of sensitivity, specificity, rapidity, and safety [(15), p. PCR based]. Conventional PCR, followed by restriction fragment length polymorphism (RFLP) or sequencing of the L1 loop region of the hexon gene, has been widely employed for FAdV genotyping (2, 16, 17). These molecular techniques have surpassed classical serological tests due to their higher sensitivity and specificity.

On the other hand, a number of studies have shown that PCR combined with high-resolution melting curve analysis (HRM) is a useful and cost-effective alternative for rapid and efficient genotyping, facilitating epidemiological investigations. This technique involves the integration of a DNA intercalating fluorochrome into the amplified DNA (18). The gradual increase in temperature at the end of PCR cycles causes denaturation of the DNA, leading to the elimination of the fluorochrome and resulting in a decrease in fluorescence. These data are recorded by a fluorescence detection system and used to generate a melting curve representing fluorescence changes based on temperature. Small variations in DNA levels result in variations in the melting temperature (Tm), thus altering the shape of the curve. By comparing the melting curve of a unknown sample with reference curves, it is possible to detect genetic variations, DNA mutations, or even molecular typing. In 2009, Steer et al. (19) established for the first time a robust closed-tube PCR-HRM genotyping technique for FAdV classification. HRM curve analysis of the PCR product generated by the HexL1-s/Hex L1-as primer pair proved to be a specific for FAdV genotyping. All serotypes generated one or more major peaks and were visually distinct from each other in their melting curve profiles, with a confidence level greater than 99%. Subsequently, the Hex L1 PCR/HRM technique was used to genotype FAdVs from 26 IBH cases originating from Australian broiler flocks, while cross-neutralization was observed between FAdV-11 and the reference serum FAdV-2 using the VN test (13).

The aim of this study is to assess the clinical, analytical, and genotyping performance of the Hex L1 PCR/HRM technique in investigating recent IBH and AGE outbreaks reported in poultry farms in four regions of Morocco. The results obtained by the Hex L1 PCR/HRM test are compared to those obtained by universal real-time PCR test targeting a conserved region of the FAdV genome (52 K gene) (20), as well as to the results obtained from sequencing of a variable region of 897 bp within the hexon gene, in order to validate the reliability and accuracy of the Hex L1 PCR/HRM technique for FAdV outbreak investigations.

## 2 Materials and methods

### 2.1 Reference FAdV strains

Representative strains of the 12 known FAdV serotypes were obtained from the University of Veterinary Medicine (Vetmeduni Vienna) as FTA cards specific to each serotype. Fragments from the impregnated sections of each cards were cut using sterile scissors, placed in Eppendorf tubes, and washed with TE buffer separately. The reference FAdV strains and their GenBank accession numbers used in this study are presented in Table 1.

**Table 1:** References strains used in this study.

Serotype	Strain name	Access number
FAdV-1	CELO	MK572875.1
FAdV-2	658	KT862806.1
FAdV-3	SR49	KT862807.1
FAdV-4	KR5	NC_075488.1
FAdV-5	340	NC_021221.1
FAdV-6	CR119	NC_038332.1
FAdV-7	YR36	KT862809.1
FAdV-8a	TR59	KT862810.1
FAdV-8b	764	KT862811.1
FAdV-9	A2-A	AF083975.2
FAdV-10	C2B	MK572851.1
FAdV-11	UF71	KT862812.1

### 2.2 Ethical statement

Tissue samples used in this study were collected from naturally dead birds. The protocol was applied in accordance with Moroccan legislation on laboratory animal care and use and animal protocols approved by the institutional Ethical Committee for Animal Veterinary Science and Public Health (CESASPV) and with international standards cited in numerous scientific references and the WOAH Manual (25).

### 2.3 Clinical samples

Organs including liver, gizzard, heart, oviduct, spleen, and kidney, were collected from broiler and layer chicken farms located in four provinces in Moroccan which is Casablanca, Rabat, Fès, and Agadir. In total, samples were collected from 20 farms, including 2 layer farms (one in Casablanca and one in Rabat) and 18 broiler farms (13 in Casablanca, 2 in Agadir, and 3 in Fès). Although the affected flocks showed no obvious clinical signs, they presented a high mortality rate (10%–15%) and typical macroscopic pathological changes in the liver, such as enlargement, discoloration, and mottling. The gizzards were often distended with fluid or hemorrhagic content and showed areas of erosion or ulceration on the koilin layer and surface mucosa.

The number of samples collected per farm was not fixed, as it depended on the number of available carcasses and the severity of the macroscopic lesions observed during necropsy. However, samples were generally collected from at least 10 carcasses per farm to ensure statistical relevance. Depending on the type and extent of the lesions, one or several organs were sampled from each bird. In most cases, tissues from each farm were pooled into a single composite. In other cases, tissues from the same farm were pooled into two to three separate composite samples representing different organs, such as gizzard, oviduct, heart, spleen + kidney (Table 2).

**Table 2:** Clinical and epidemiological information of the 26 field samples included in this study.

Field samples ID	Year	Origin	Spices	Sample type	Species	Age (days)
1,239-2	2023	Casablanca	Layer	Gizzard	Layers	162
1,239-3	2023	Casablanca	Layer	Oviduct	Layers	162
1,240	2023	Rabat	Layer	Liver + Gizzard	Layers	588
276-1	2024	Casablanca	Broiler	liver	Broilers	20
337	2024	Casablanca	Broiler	liver	Broilers	15
338	2024	Casablanca	Broiler	Liver + Heart	Broilers	14
339-2	2024	Casablanca	Broiler	Gizzard	Broilers	18
339-3	2024	Casablanca	Broiler	Heart	Broilers	18
339-4	2024	Casablanca	Broiler	Spleen + kidney	Broilers	18
340-2	2024	Casablanca	Broiler	Gizzard	Broilers	18
340-3	2024	Casablanca	Broiler	Heart	Broilers	18
341-4	2024	Casablanca	Broiler	Spleen + kidney	Broilers	14
344-1	2024	Fès	Broiler	Liver	Broilers	14
346-2	2024	Fès	Broiler	Heart	Broilers	14
349-2	2024	Fès	Broiler	Heart	Broilers	14
618-3	2023	Casablanca	Broiler	Gizzard	Broilers	40
352-1	2024	Casablanca	Broiler	Heart	Broilers	14
352-2	2024	Casablanca	Broiler	Gizzard	Broilers	14
793	2023	Casablanca	Broiler	Gizzard	Broilers	43
658	2023	Agadir	Broiler	Gizzard	Broilers	17
669	2023	Agadir	Broiler	Gizzard	Broilers	38
OI2	2019	Rabat	Broiler	Liver	Broilers	35
P2	2015	Casablanca	Broiler	Liver	Broilers	22
P3	2015	Casablanca	Broiler	Liver	Broilers	22
OI5	2023	Casablanca	Broiler	Liver	Broilers	37
P1	2015	Casablanca	Broiler	Liver	Broilers	22

Samples highlighted in the same color originate from the same farm.

The collected tissues were homogenized and processed for viral DNA extraction, following the protocol described by Abghour et al. (5) and Ouchhour et al. (21) for deep molecular characterization.

### 2.4 Viral DNA extraction

Viral DNA from FAdV reference strains was extracted by the MagPurix EVO Automated Nucleic Acid Purification System (Zinexts, Taiwan) using the MagPurix^®^ Viral/Pathogen Nucleic Acid Extraction Kit, according to the manufacturer’s protocol. An endogenous control was included during the extraction process to monitor the efficiency and integrity of nucleic acid recovery. For the 26 field samples, nucleic acids were extracted using the Kylt^®^ RNA/DNA Purification Kit (Hoeltinghausen, Germany) according to the manufacturer’s instructions. The same extraction kit and protocol were applied uniformly to all 26 samples.

### 2.5 Conventional PCR

DNA extracted from field samples was amplified using universal primers Hexon A/Hexon B. These primers target the hexon gene, producing an amplicon of approximately 897 bp (2). Amplification was carried out with the DreamTaq Green PCR Master Mix (2X) kit (Thermo Fisher Scientific, Waltham, USA). The 45 μL reaction mixture included 5 μL of DNA sample, 25 μL of Master Mix, 2 μL of each primer at 10 μM, and the volume was completed with PCR grade water. Thermal cycling conditions for conventional PCR consisted of an initial denaturation at 95 °C for 5 min, followed by 45 cycles with denaturation at 95 °C for 30 s, annealing at 62 °C for 30 s, and extension at 72 °C for 1 min, with a final extension at 72 °C for 10 min.

Position is given for the FAdV-1 CELO complete genome (GenBank accession number: U46933).

### 2.6 DNA sequence analysis

The amplified PCR products using Hexon A/Hexon B primers were analyzed by 1.5% agarose gel electrophoresis in Tris-borate-EDTA buffer and stained with ethidium bromide (Promega, Madison, USA). Positive samples were purified, and subjected to *Sanger sequencing*. The resulting sequences were compared with available sequences in GenBank using Basic Local Alignment Search Tool (BLAST)^1^ provided by the National Center for Biotechnology Information (NCBI).

The obtained sequences, along with reference strains retrieved from GenBank, were aligned using ClustalW version 2.1^2^ (22), and pairwise sequence identity and similarity were calculated using the SIAS online tool.^3^ A phylogenetic tree was constructed in Geneious Prime version 2024.0.7 using the Neighbor-Joining (NJ) method. A table listing the reference strains used in the phylogenetic analysis along with their GenBank accession numbers has been added as supplementary material.

### 2.7 Real-time PCR

The DNA extracted from field samples was first amplified by SYBR Green universal real-time PCR/52 K as a reference test using 52 K-fw (sense primer) and 52 K-rv (antisense primer) following the protocol described by Günes et al. (20). Real-time PCR was performed on a QuantStudio 5 Real-Time PCR System (Thermo Fisher Scientific, USA). Each reaction consisted of 5 μL of DNA and 15 μL of a reaction mix containing 10 μL of the SensiFAST SYBR Lo-Rox kit (Bioline, New York, NY, USA), which includes Taq DNA polymerase, SYBR Green I, and a deoxynucleotide triphosphates (dNTPs) mix, along with 0.8 μL of each primer (52 K-fw/52 K-rv) at a concentration of 10 μM, and 3.4 μL of PCR-grade water.

### 2.8 Assessment of intra- and inter-assay variability

To evaluate the reproducibility and repeatability of the Hex L1 PCR/HRM assay, four DNA concentrations ranging from log₁₀(2.2) to log₁₀(9) IU/mL were tested in triplicate over three consecutive days. Intra-assay variability was determined by analyzing replicate measurements within the same run, whereas inter-assay variability was assessed by comparing results obtained on different days. Coefficients of variation (CV) were calculated from the mean and standard deviation of the Ct values.

### 2.9 Analytical performance of Hex L1 PCR/HRM

To evaluate the analytical performance of the Hex L1 PCR/HRM assay, a positive FAdV control corresponding to FAdV-1 was provided by AVSBIO and used for the analytical performance analysis. Upon receipt, the concentration of the positive control was quantified using a Qubit Fluorometer (Thermo Fisher Scientific) using Qubit^™^ dsDNA Quantification Assay Kits (Thermo Fisher, Invitrogen) to ensure accuracy before testing. A series of tenfold dilutions was then prepared from this stock solution, ranging from Log₁₀ (2.2) to Log₁₀ (9) IU/mL. Each dilution was amplified in triplicate using the Hex L1 PCR/HRM assay on three successive days to assess both intra- and inter-assay variability.

### 2.10 Hex L1 PCR/HRM analysis

Amplification of approximately 590 base pair (bp) region of the hexon gene using degenerated primers (Hex L1-s and Hex L1-as), was performed on LightCycler 480 (Roche Diagnostics, USA). Each reaction consisted of 5 μL of DNA and 15 μL of a reaction mix containing 5 μL of the Takyon Low ROX SYBR 2X Master Mix includes Taq DNA polymerase, SYBR Green I, and a dNTPs mix, along with 1 μL of each primer (HexL1/Hex L1s) at a concentration of 5 μM and 7 μL of PCR-grade water to adjust the total volume to 20 μL. The reaction mixtures were subjected to 94 °C for 2 min, and then 40 cycles of 94 °C for 20 s, 56 °C for 20 s, and 72 °C for 30 s. Optical measurements in green channel (excitation at 470 nm and detection at 510 nm) were recorded during the extension step. After PCR cycles, a final extension of 72 °C for 2 min was performed. The HRM curve analysis was performed following the protocol described by Steer et al., using LightCycler software, version 1.5.0, and the HRM algorithm.

The conventional melt curves were generated automatically by LightCycler software. To generate the normalized HRM curves, normalization regions were applied as follows: 81.5–82.0 °C and 90.0–90.5 °C. Genotypes were defined by using a representative strain for each serotype (FAdV reference strain in our case). The software then auto-assigned the genotype to each tested sample and provided confidence percentages (C%). No confidence threshold was applied to the analysis, allowing for the visualization of all assigned genotypes and their respective C%. All clinical samples were tested in triplicate to detect variations induced by technical errors. The means of the C% of the sample replicates correctly assigned to a representative genotype, along with the standard deviation (SD) and standard error (SE), were calculated using Excel.

## 3 Results

### 3.1 Clinical and histopathological characterization of IBH and AGE field cases

Twenty-six clinical samples (liver, gizzard, heart, spleen + kidney, and oviduct) from broiler and layer flocks were collected from 20 farm located in four provinces in Morocco between 2015 and 2024 (Table 2) and analyzed at the Molecular Biology Laboratory, Avian Pathology Unit, Hassan II Agronomy and Veterinary Institute. Flocks showed increased mortality (10%–15%) without typical clinical signs.

Inclusion body hepatitis and adenoviral gizzard erosion were confirmed through histopathological examination, which revealed characteristic lesions for both diseases. IBH cases showed mild to severe mononuclear inflammatory cell infiltration in the liver and basophilic intranuclear inclusion bodies in degenerated hepatocytes, confirming FAdV infection. AGE cases showed mild to severe epithelial necrosis, surface erosions or ulcers, and inflammatory cell infiltration in the gizzard, with eosinophilic intranuclear inclusion bodies occasionally observed in the epithelial cells. However, two samples (1,240 and 340-2) originating from two distinct farms showed no FAdV-specific lesions upon histological examination.

Among the 26 field samples, 25 (96.15%) tested positive by conventional PCR (cPCR), showing distinct bands on agarose gel. However, one sample (3.84%) tested negative, with no visible bands. Notably, this sample also showed no FAdV-specific lesions upon histopathological examination, which highlights the correlation between the cPCR and histopathological results (see Table 3).

**Table 3:** Sequences of the primers used in this study.

Primer name	Position	Sequence (5′….0.3′)	Reference
Hexon A	18,432–18,449	CAA RTT CAGRCA GAC GGT	Meulemans et al. (2)
Hexon B	19,367–19,346	TAG TGA TGM CGS GAC ATC AT	
52 K-fw	13,075–13,093	ATGGCK CAG ATG GCY AAG G	Günes et al. (20)
52 K-rv	13,250–13,232	AGC GCC TGG GTC AAACCG A	
Hex L1-s	18,589–18,612	ATGGGAGCSACCTAYTTCGACAT	Steer et al. (19)
Hex L1-as	19,202–199,180	AAATTGTCCCKRAANCCGATGTA	

### 3.2 Sequencing and phylogenetic analysis

**Figure 1:**
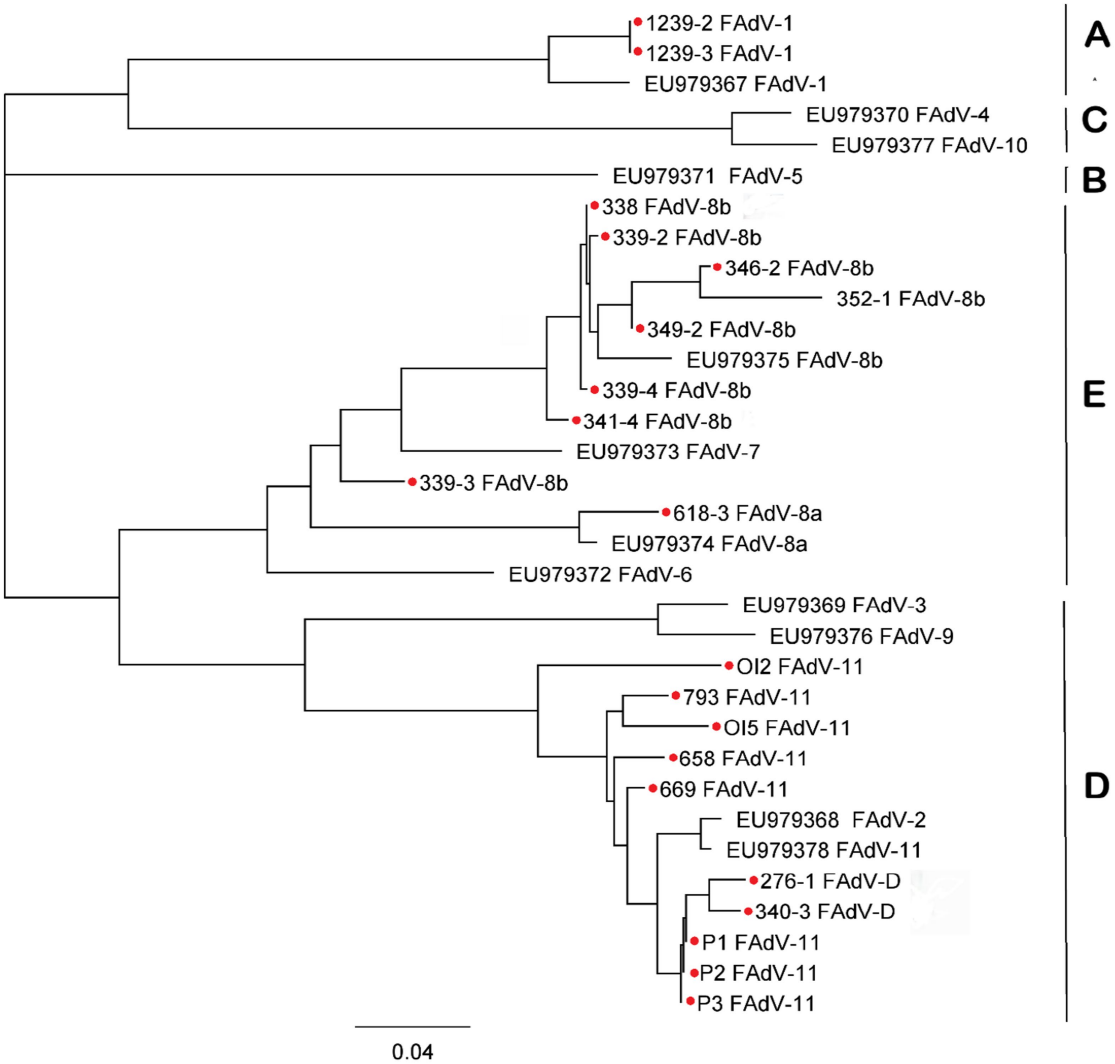
Phylogenetic tree based on loop 1 region (Hexon gene) PCR product sequences, illustrating FAdV serotypes grouped into the five known species (FAdV-A to FAdV-E). Field samples (marked with a red dot) clustered within three species: FAdV-A (serotype 1), FAdV-D (serotype 11), and FAdV-E (serotypes 8a and 8b). The tree was constructed using the neighbor-joining (NJ) method in Geneious Prime version 2024.0.7.

Of the 26 field samples, only 22 were sequenced. DNA sequencing results identified four circulating FAdV serotypes. Among the IBH cases, FAdV-11 (8/22), FAdV-D (2/22), and FAdV-8b (9/22) were detected, while in AGE cases, FAdV-1 (2/22) and FAdV-8a (1/22) were identified. The corresponding GenBank accession numbers for the hexon gene sequences of these samples are listed in Table 4. Phylogenetic analysis of the Hex L1 region, including these field isolates and 12 reference strains, showed that all reference strains clustered according to their representative species, as illustrated in the phylogenetic tree (Figure 1).

**Table 4:** Field samples used in this study, their PCR results, sequencing, and HRM analysis results.

Field samples ID	Histopathologic examination results	Ct (real-time PCR/52 K test)	Mean Ct (HexL1) (PCR/HRM)	Conventional PCR results	Hexon sequencing results	Hex L1 PCR/HRM results	Access number
1,239-2	GE	17.58	18.42	+	FAdV A-1	FAdV A-1	PP860913
1,239-3	GE	21.6	25.69	+	FAdV A-1	FAdV A-1	PP860914
1,240	--	26.99	31	−	ND	FAdV-D	ND
276-1	IBH	9.99	9.12	+	FAdV D	FAdV D	PQ311676
337	IBH	4.85	6.43	+	FAdV-8b	FAdV-8b	PQ324696
338	IBH	13.15	14.97	+	FAdV E-8b	FAdV E-8b	PQ324695
339-2	IBH	16.04	17	+	FAdV E-8b	FAdV E-8b	PQ324696
339-3	IBH	15.98	15.46	+	FAdV E-8b	FAdV E-8b	PQ324697
339-4	IBH	11.96	12	+	FAdV E-8b	FAdV E-8b	PQ324698
340-2	--	11.61	15.51	+	ND	FAdV-D	ND
340-3	IBH	12.18	17.35	+	FAdV D	FAdV D	PQ311677
341-4	IBH	11.52	9.45	+	FAdV E-8b	FAdV E-8b	PQ324700
344-1	IBH	4.72	10,58	+	ND	FAdV-11	ND
346-2	IBH	15.14	13.76	+	FAdV E-8b	FAdV E-8b	PQ324703
349-2	IBH	13.32	19.08	+	FAdV E-8b	FAdV E-8b	PQ324707
618-3	GE	26	27.24	+	FAdV E-8a	FAdV E-8a	PP429842
352-1	IBH	14.73	16.26	+	FAdV E-8b	FAdV E-8b	PQ324710
352-2	IBH	14.41	13.95	+	ND	FAdV-11	ND
793	IBH	26.91	25.75	+	FAdV D-11	FAdV D-11	PP798952
658	IBH	23.48	25.38	+	FAdV D-11	FAdV D-11	PP798942
669	IBH	11.29	11.91	+	FAdV D-11	FAdV D-11	PP798944
OI2	IBH	29.24	30.6	+	FAdV D-11	FAdV D-11	PP798947
P2	IBH	23.45	24.83	+	FAdV D-11	FAdV D-11	MK468900
P3	IBH	21.46	22.76	+	FAdV D-11	FAdV D-11	MK468899
OI5	IBH	26.68	27.63	+	FAdV D-11	FAdV D-11	PP798949
P1	IBH	25.61	25.21	+	FAdV D-11	FAdV D-11	MK468898

FAdV, fowl adenovirus; HRM, high-resolution melt; IBH, inclusion body hepatitis; --, not specific FAdV inclusion; ND, not determined.

Sequence identity analysis, performed using ClustalW and SIAS,^4^ revealed varying levels of similarity between the field samples and their corresponding reference strains (see Supplementary material). Isolates identified as FAdV-11 showed sequence identity ranging from 88.53% to 91.73% with the FAdV-11 reference strain 380. The FAdV-1 sample exhibited 86.61% identity with the CELO reference strain, while the FAdV-8a sample showed 90.1% identity with the TR59 reference strain. For FAdV-8b, eight out of nine field samples displayed sequence identities ranging from 74.53% to 84.73% with the reference strain 764. However, one field sample (339-3) showed markedly lower identity, with only 57.73% similarity to with FAdV-8b reference strain and 57% with FAdV-7, suggesting a higher genetic divergence. Finally, two isolates classified as FAdV-D displayed identity values of 84.14% and 85.16% with the FAdV-11 reference strain, and 85.16% and 85.32% with the FAdV-2 reference strain, respectively. These results highlight the diversity among field samples and underline the importance of sequence analysis for accurate serotype identification.

### 3.3 Analytical and clinical performance of the Hex L1 PCR/HRM test

#### 3.3.1 Inter- and intra-assay variation

The Hex L1 PCR/HRM assay demonstrated excellent reproducibility and repeatability. As shown in Table 5. As shown in Table 5, the assay showed intra- and inter-assay coefficients of variation ranging from 0.19% to 0.70% and 0.18% to 1.82%, respectively, indicating excellent reproducibility and repeatability.

**Table 5:** Repeatability and Reproducibility of Hex L1 PCR/HRM assay.

DNA concentration (log_10_ IU/mL)	Intra-assay variability			Inter-assay variability		
	Mean Ct	SD	CV (%)	Mean Ct	SD	CV (%)
2.2	27.24	0.054	0.19	27.44	0.29	1.09
5.3	22.42	0.10	0.48	22.52	0.04	0.18
7	20.51	0.053	0.26	20.46	036	1.79
9.1	10.58	0.074	0.70	6.536	0.11	1.82

IU, international unit; Ct, cycle threshold; SD, standard deviation; CV, coefficient of variation.

#### 3.3.2 Concordance with real-time PCR/52 K assay

**Figure 2:**
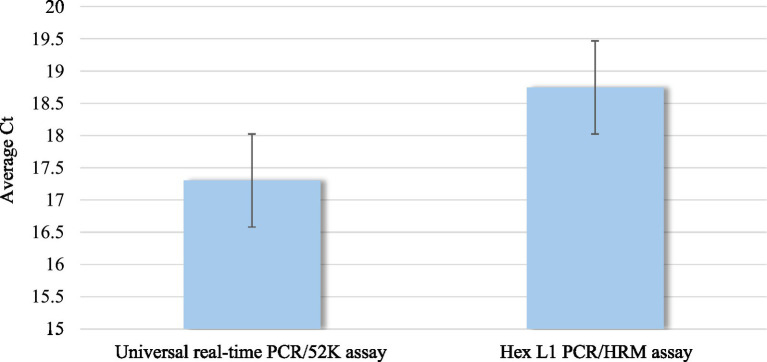
Comparison of mean Ct obtained by Hex L1 PCR/HRM assay and universal real-time PCR/52 K test.

FAdV was detected in all 26 tested samples (100%) by both the Hex L1 PCR/HRM assay and the universal real-time PCR/52 K test. The average Ct value of all tested samples was 18.70 for the Hex L1 PCR/HRM assay, which was slightly higher than that obtained by the universal real-time PCR/52 K assay (17.30). However, this difference was not statistically significant (*p* < 0.001) (Figure 2).

**Figure 3:**
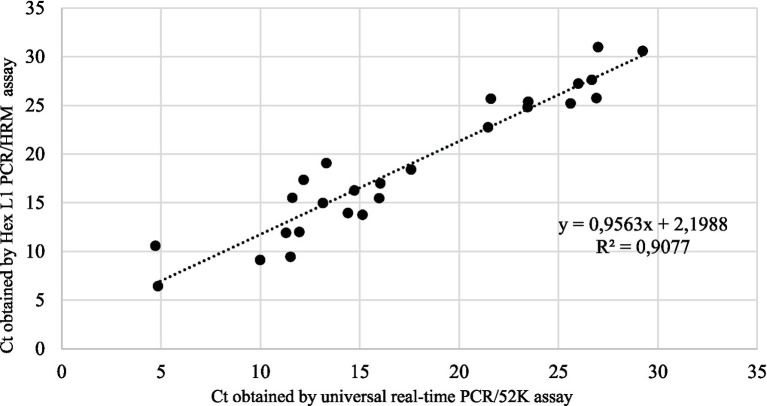
Linear regression analysis of correlation between Hex L1 PCR/HRM test and universal real-time PCR/52 K test.

The overall correlation between the universal real-time PCR/52 K test and Hex L1 PCR/HRM test for all samples tested (*n* = 26) gave a linear equation of *y = 0.9563x + 2.1988* with a coefficient of correlation (*R*^2^) of *0.9077*, indicating a strong and significant correlation between the two tests (Figure 3).

#### 3.3.3 Comparative turnaround time

In terms of turnaround time, the Hex L1 PCR/HRM assay is considerably faster than cPCR followed by DNA sequencing and also faster than the real-time PCR/52 K assay, which requires approximately 60 min for amplification. The Hex L1 PCR/HRM assay requires about 49 min for amplification plus 2 min for melting curve generation, enabling simultaneous detection and genotyping in a single run. In contrast, cPCR requires approximately 105 min for amplification plus an additional 40 min for electrophoresis, followed by multiple downstream steps for sequencing, which significantly increases the total time for obtaining the result.

### 3.4 Genotyping performance of Hex L1 PCR/HRM assay

**Figure 4:**
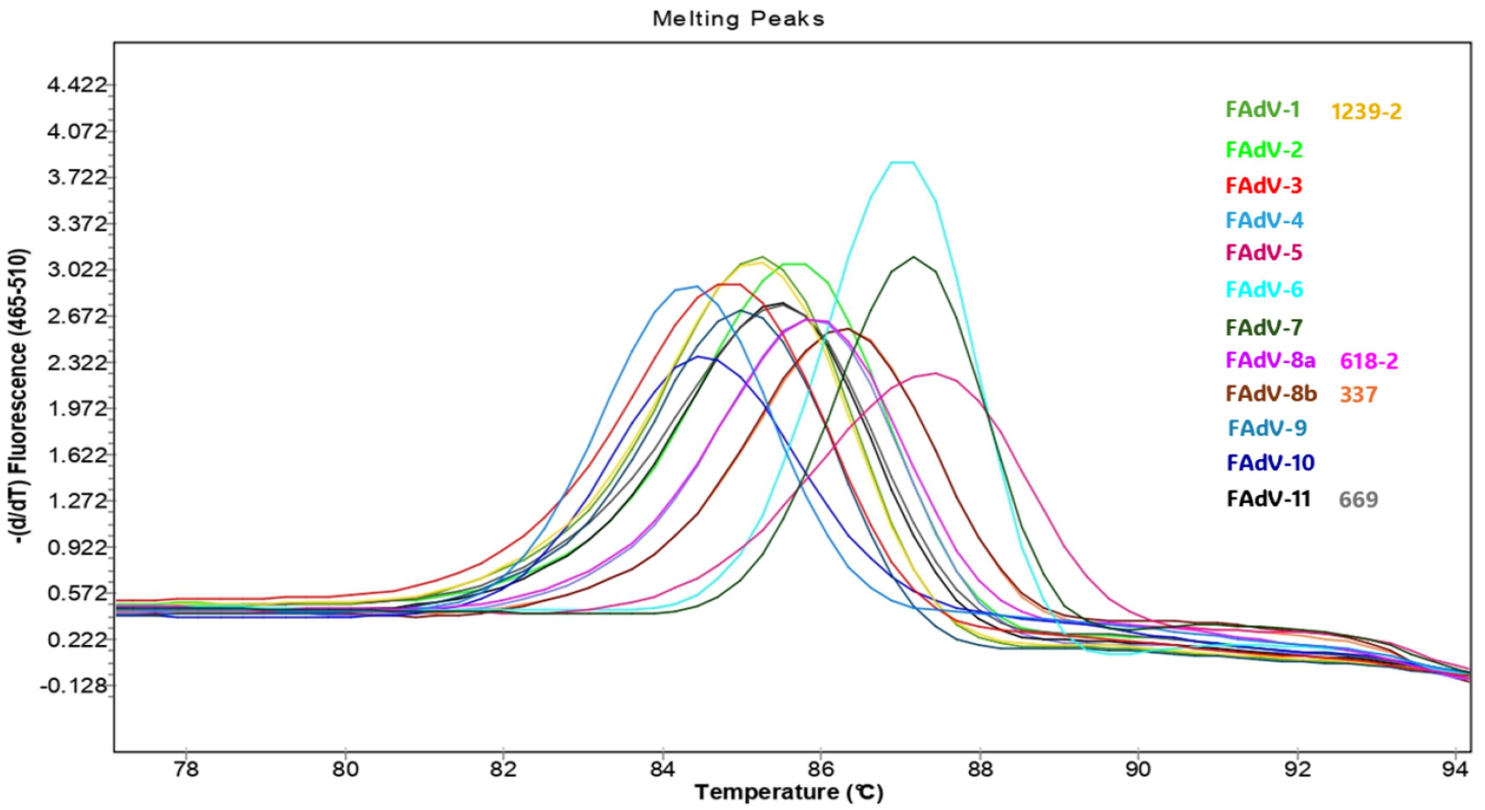
Conventional melting curves of the hexon gene (loop 1 region) PCR products from 12 FAdV reference serotypes and four Moroccan field isolates representing four serotypes, with each field isolate labeled next to its corresponding reference strain of the same serotype.

To assess the reliability of HRM curve analysis for FAdV genotyping, the results were compared to Hex L1 sequencing, considered as a reference method for FAdV genotyping. Twelve reference strains representing the 12 FAdV genotypes were used, along with 26 field sample from IBH and GE cases. Each sample was tested in triplicate to evaluate result reproducibility. Four samples, representing four distinct serotypes, were selected to illustrate the results (Figure 4).

The HRM curve analysis obtained from 12 reference strains and four field samples (669, 618-2, 337, and 1,239-2) revealed 12 distinct melting profiles (Figure 4), each with a single peak. The melting temperatures of the 12 genotypes ranged from 84.3 °C to 87.5 °C. FAdV-4, belonging to species B, exhibited the lowest Tm (84.3 °C), whereas FAdV-5 (species C) had the highest Tm (87.5 °C). Some serotypes showed minimal temperature differences of approximately 0.3 °C, such as FAdV-10 with FAdV-4, FAdV-9 with FAdV-3, FAdV-11 with FAdV-2, and FAdV-6 with FAdV-7. Although FAdV-6 and FAdV-7 exhibited nearly identical Tm values, their melting curve shapes were distinct, allowing reliable differentiation between the two serotypes. Field samples identified as FAdV-8b by sequencing displayed peaks between 86.7 °C and 87.0 °C. Samples genotyped as FAdV-1 exhibited peaks ranging from 84.7 °C to 85.6 °C, while those classified as FAdV-11 had peaks between 85.7 °C and 85.7 °C. Finally, sample 618-2, identified as FAdV-8a, showed a single peak at 86 °C. Representative samples for FAdV-1, FAdV-8a, FAdV-8b, and FAdV-11 (1239-2, 618-2, 337, and 669, respectively) displayed HRM profiles that perfectly matched those of their corresponding reference serotypes.

The normalization of HRM curves data (Data not shown) confirmed the genotyping results. Isolates identified as FAdV-8b showed an average similarity coefficient (C%) of 82.1 ± 4.2% with the FAdV-8b reference strain. When this reference strain was removed, these isolates were then classified as FAdV-7 with an average C% of 40.3 ± 5.9%. The isolate initially genotyped as FAdV-8a showed an average C% of 81% when compared to the reference strains. Similarly, FAdV-11 isolates displayed an average C% of 81%. In the absence of the FAdV-11 reference strain, isolates were assigned to genotype FAdV-2 with a C% of 44.0 ± 1.6%. Finally, isolates identified as FAdV-1 also showed an average similarity of 81.2 ± 2% with FAdV-1 reference strain.

## 4 Discussion

The objective of this study is to assess the clinical, analytical, and genotyping performance of the Hex L1 PCR/HRM technique in investigating recent IBH and AGE outbreaks reported in 20 poultry farms (2 layer, and 18 broiler) across 4 regions of Morocco. The results obtained by the Hex L1 PCR/HRM test are compared to those obtained by a real-time PCR test, as well as the sequencing results of Loop 1 region within Hexon FAdV gene, in order to validate the reliability and accuracy of the Hex L1 PCR/HRM technique for FAdV outbreak investigations in Morocco.

In this study, FAdV-8b, FAdV-D, and FAdV-11 were identified in samples from 16 broiler farms showing characteristic IBH lesions, as well as from two broiler farms without IBH-specific lesions. This aligns with previous findings in Morocco (5, 23), which also reported FAdV-8b and FAdV-11, with FAdV-11 being the most prevalent in IBH cases. Regarding AGE, FAdV-8a was detected in one broiler farm, and FAdV-1 was identified in a layer farm with both farms showing AGE lesions. These findings align with previous reports (21), which described the involvement of FAdV-1 and FAdV-8a in AGE cases in Morocco. Overall, these results highlight the strong correlation between histopathological and sequencing findings.

Sequence identity analysis, performed using ClustalW and SIAS (Sequence Identity and Similarity), revealed varying degrees of similarity between field samples and their respective reference strains. Notably, sample 339-3, genotyped as FAdV-8b, exhibited low genetic similarity (57.63%) with strain 764 (representing FAdV-8b) and 57% with strain YR36 (representing FAdV-7). This genetic divergence suggests the possible presence of a recombinant strain or a distinct genetic variant. These findings highlight the importance of continuous surveillance to identify emerging viral variants, which could potentially impact current control and vaccination strategies. These findings align with the observations of reported by Schachner et al. (24), who demonstrated that the most recombination events identified among FAdVs occurred within species E, predominantly in isolates associated with IBH cases.

The Hexon L1 PCR/HRM technique was developed as a rapid method for the detection and differentiation of all 12 FAdV serotypes (13, 19). The Hexon L1 PCR/HRM assay was able to detect all 12 FAdV reference serotypes, confirming its universality through the use of degenerate primers that enable amplification despite genetic variations between serotypes. Analytical performance assessment further demonstrated excellent reproducibility and repeatability, with intra-assay CV values ranging from 0.19% to 0.70%, and inter-assay CV values between 0.18% and 1.82%. These results indicate a high level of precision and reliability of the test for FAdV detection. Furthermore, the assay showed strong correlation with the real-time PCR/52 K assay, with an *R*^2^ value of 0.9077. This high correlation confirms that the Hexon L1 PCR/HRM assay is equally effective as the real-time PCR/52 K test for the detection of FAdVs.

On the other hand, the clinical performance results showed that the Hex L1 PCR/HRM assay was more sensitive than cPCR using Hexon A/Hexon B primers. While 100% of the samples (26/26) were detected by both the real-time PCR/52 K assay and the Hex L1 PCR/HRM assay, only 96% (25/26) were detected by cPCR. This sample (ID: 1240) showed one of the highest Ct values among all samples (Ct = 26.99 by the real-time PCR/52 K, and Ct = 31 by the Hex L1 PCR/HRM assay). This relatively low viral concentration likely explain its negative results by cPCR. In this context, (20) reported that cPCR (Hexon A/Hexon B) is 10 times less sensitive than real-time PCR/52 K assay, which supports our findings. Nevertheless, this sample was successfully detected and genotyped by the Hex L1 PCR/HRM as FAdV-D, highlighting the sensitivity of this technique.

In terms of turnaround time, the Hex L1 PCR/HRM assay offers a clear advantage by enabling rapid detection and genotyping within the same reaction. While the real-time PCR/52 K assay requires approximately 60 min for amplification, the Hex L1 PCR/HRM assay completes detection and genotyping in about 51 min, resulting in a shorter overall processing time. In contrast, conventional PCR requires about 105 min for amplification, followed by an additional 40 min for electrophoresis and further downstream steps for sequencing, which substantially prolong the time to result. Although the Hex L1 PCR/HRM assay is faster and simpler, conventional PCR coupled with sequencing remains the reference method for FAdV genotyping, and sequencing validation is still necessary to confirm HRM-based results.

Regarding genotyping performance, the analysis of the Hex L1 PCR/HRM melting curve for the 12 reference FAdV strains showed a single peak for each strain. This contrasts with other studies, such as those by Steer et al. (13, 19), which reported two peaks for some serotypes as FAdV-6, FAdV-7, FAdV-8b, and FAdV-11, and three peaks for FAdV-8a. The discrepancy observed for FAdV-11 may be explained by the use of different reference isolates (UF-71 in the cited study versus 380 in our study), as isolate-specific polymorphisms within the target region can affect thermal stability and produce multiple melting transitions. For the other FAdV serotypes that showed more than one peak, this difference may be attributable to analytical factors rather than biological variation. Variables such as the use of different PCR master mixes, variations in PCR cycling programs, the type and concentration of the DNA-binding dye, reaction chemistry, Mg^2+^ and salt concentrations, DNA input amounts, ramp rate, and the thermocycler platform can all influence melting behavior and may either merge closely spaced transitions into a single peak or resolve them as multiple peaks during HRM analysis.

The Hex L1 PCR/HRM assay successfully genotyped all field samples, with results consistent with sequencing outcomes, confirming its effectiveness as a rapid tool for FAdV detection and genotyping. However, some challenges were noted in distinguishing closely related serotypes within the same species, particularly FAdV-2 and FAdV-11 (species D), FAdV-3 and FAdV-9 (species D), FAdV-7 and FAdV-6 (species E), as well as FAdV-4 and FAdV-10 (species C). These difficulties are likely due to their high genetic similarity, with similarity rates of 97.79%, 92.82%, 84.82%, and 93.25% respectively, as calculated using the SIAS online tool, which may explain their closely overlapping melting temperatures in HRM analysis. In this context, four field samples exhibited similar Tm values and HRM profiles that completely overlapped with those of FAdV-11 and FAdV-2. Consequently, these samples were classified as FAdV-D. The same issue was encountered during DNA sequencing, further reinforcing the challenge of achieving precise serotype-level resolution among closely related serotypes within the same species. Therefore, further validation using well-characterized field samples representing the remaining serotypes (FAdV-4, FAdV-2, FAdV-7, FAdV-3, and FAdV-9) is recommended to strengthen the evaluation of the clinical performance of the Hex L1 PCR/HRM assay for genotyping FAdV field strains.

In addition to the cited limitation, the method relies only on characteristic melting profiles for genotype identification. Any sequence variation or mutation in the target region could shift the melting peak and lead to misclassification of FAdV variants. Therefore, ongoing DNA sequencing is essential to track genetic diversity and confirm HRM results. Also, because relies on intercalating dyes, non-specific amplicons can reduce specificity if present. These limitations highlight the need to regularly use sequencing to validate HRM results.

## 5 Conclusion

In conclusion, the Hex L1 PCR/HRM method offers a fast, sensitive, and reliable alternative for detecting and genotyping FAdV. It provides universal detection, quantification, and genotyping in a single step, surpassing the limitations of traditional methods. However, distinguishing some serotypes within the same species remains challenging, necessitating sequencing for validation. Overall, the method can be effectively used for screening but requires more accurate techniques, such as sequencing, to confirm genotyping results. Additionally, validation using a broader range of clinical isolates representing other FAdV serotypes is recommended to further assess the method’s performance across diverse FAdV strains.

## Data Availability

The datasets presented in this study can be found in online repositories. The names of the repository/repositories and accession number(s) can be found in the article/Supplementary material.

## Footnotes

1
https://blast.ncbi.nlm.nih.gov/Blast.cgi

2
https://www.ebi.ac.uk/Tools/msa/clustalw2

3
http://imed.med.ucm.es/Tools/sias.html

4
http://imed.med.ucm.es/Tools/sias.html

## Supplementary material

SUPPLEMENTARY FIGURE S1 Multiple sequence alignment of the forward primers (HEXON A and Hex L1) used in this study, shown across five FAdV serotypes corresponding to the five recognized species.

Supplementary FIGURE S2 Multiple sequence alignment of the reverse primers (HEXON B and Hex L1 as) used in this study, shown across five FAdV serotypes corresponding to the five recognized species.

## References

[1] Hess M. Detection and differentiation of avian adenoviruses: a review. Avian Pathol. (2000) 29:195–206. doi: 10.1080/03079450050045440, PMID: 19184805

[2] Meulemans G Boschmans M Berg TP Decaesstecker M. Polymerase chain reaction combined with restriction enzyme analysis for detection and differentiation of fowl adenoviruses. Avian Pathol J WVPA. (2001) 30:655–60. doi: 10.1080/03079450120092143, PMID: 19184959

[3] Thanasut K Fujino K Taharaguchi M Taharaguchi S Shimokawa F Murakami M . Genome sequence of fowl aviadenovirus a strain JM1/1, which caused gizzard erosions in Japan. Genome Announc. (2017) 5:e00749. doi: 10.1128/genomea.00749-17, PMID: 29025927 PMC5637487

[4] Alvarado IR Villegas P El-Attrache J Jensen E Rosales G Perozo F . Genetic characterization, pathogenicity, and protection studies with an avian adenovirus isolate associated with inclusion body hepatitis. Avian Dis. (2007) 51:27–32. doi: 10.1637/0005-2086(2007)051[0027:GCPAPS]2.0.CO;2, PMID: 17461263

[5] Abghour S Zro K Mouahid M Tahiri F Tarta M Berrada J . Isolation and characterization of fowl aviadenovirus serotype 11 from chickens with inclusion body hepatitis in Morocco. PLoS One. (2019) 14:e0227004. doi: 10.1371/journal.pone.0227004, PMID: 31891942 PMC6938405

[6] Domanska-Blicharz K Tomczyk G Smietanka K Kozaczynski W Minta Z. Molecular characterization of fowl adenoviruses isolated from chickens with gizzard erosions. Poult Sci. (2011) 90:983–9. doi: 10.3382/ps.2010-01214, PMID: 21489943

[7] Schachner A Matos M Grafl B Hess M. Fowl adenovirus-induced diseases and strategies for their control—a review on the current global situation. Avian Pathol. (2018) 47:111–26. doi: 10.1080/03079457.2017.1385724, PMID: 28950714

[8] Nakamura K Tanaka H Mase M Imada T Yamada M. Pancreatic necrosis and ventricular Erosion in adenovirus-associated hydropericardium syndrome of broilers. Vet Pathol. (2002) 39:403–6. doi: 10.1354/vp.39-3-403, PMID: 12014508

[9] Pathak RC Singh CM. A preliminary report on the isolation and identification of pleuropneumonia-like organisms from poultry*. Poult Sci. (1959) 38:956–9. doi: 10.3382/ps.0380956

[10] Balamurugan V Kataria JM. The hydropericardium syndrome in poultry—a current scenario. Vet Res Commun. (2004) 28:127–48. doi: 10.1023/b:verc.0000012115.86894.1e, PMID: 14992243

[11] Berg NW. Rapid detection of infectious bursal disease antibodies by counterimmunoelectrophoresis. Avian Pathol. (1982) 11:611–4. doi: 10.1080/03079458208436136, PMID: 18770227

[12] Kumar R Chandra R Shukla SK Agrawal DK Kumar M. Hydropericardium syndrome (HPS) in India: a preliminary study on the causative agent and control of the disease by inactivated autogenous vaccine. Trop Anim Health Prod. (1997) 29:158–64. doi: 10.1007/BF02633014, PMID: 9316232

[13] Steer P O’Rourke D Ghorashi S Noormohammadi A. Application of high-resolution melting curve analysis for typing of fowl adenoviruses in field cases of inclusion body hepatitis. Aust Vet J. (2011) 89:184–92. doi: 10.1111/j.1751-0813.2011.00695.x 21495991

[14] Erny K Pallister J Sheppard M. Immunological and molecular comparison of fowl adenovirus serotypes 4 and 10. Arch Virol. (1995) 140:491–501. doi: 10.1007/BF01718426, PMID: 7733822

[15] Mirmajlessi SM Destefanis M Gottsberger RA Mänd M Loit E. PCR-based specific techniques used for detecting the most important pathogens on strawberry: a systematic review. Syst Rev. (2015) 4:9. doi: 10.1186/2046-4053-4-9, PMID: 25588564 PMC4320524

[16] Ojkić D Krell PJ Tuboly T Nagy E. Characterization of fowl adenoviruses isolated in Ontario and Quebec, Canada. Can J Vet Res = Revue Canadienne De Recherche Veterinaire. (2008) 72:236–41. PMID: 18505186 PMC2327250

[17] Schachner A Marek A Grafl B Hess M. Detailed molecular analyses of the hexon loop-1 and fibers of fowl aviadenoviruses reveal new insights into the antigenic relationship and confirm that specific genotypes are involved in field outbreaks of inclusion body hepatitis. Vet Microbiol. (2016) 186:13–20. doi: 10.1016/j.vetmic.2016.02.008, PMID: 27016752

[18] Herrmann MG Durtschi JD Bromley LK Wittwer CT Voelkerding KV. Amplicon DNA melting analysis for mutation scanning and genotyping: cross-platform comparison of instruments and dyes. Clin Chem. (2006) 52:494–503. doi: 10.1373/clinchem.2005.063438, PMID: 16423901

[19] Steer PA Kirkpatrick NC O’Rourke D Noormohammadi AH. Classification of fowl adenovirus serotypes by use of high-resolution melting-curve analysis of the hexon gene region. J Clin Microbiol. (2009) 47:311–21. doi: 10.1128/jcm.01567-08 19036935 PMC2643661

[20] Günes A Marek A Grafl B Berger E Hess M. Real-time PCR assay for universal detection and quantitation of all five species of fowl adenoviruses (FAdV-A to FAdV-E). J Virol Methods. (2012) 183:147–53. doi: 10.1016/j.jviromet.2012.04.005, PMID: 22561984

[21] Ouchhour I Fellahi S Arbani O Mastour M Achehal El Kadmiri A Mouahid M . Gizzard erosion and ulceration syndrome in Moroccan poultry flocks and molecular characterization of fowl adenoviruses (FAdV). Avian Dis. (2024) 68:217–24. doi: 10.1637/aviandiseases-D-24-00004, PMID: 39400216

[22] Larkin MA Blackshields G Brown NP Chenna R McGettigan PA McWilliam H . Clustal W and Clustal X version 2.0. Bioinformatics. (2007) 23:2947–8. doi: 10.1093/bioinformatics/btm404, PMID: 17846036

[23] Redondo H Fragoso JS Tahala MA Bensassi Y Gil I Elbachir E . Characterization of strain of fowl adenoviruses circulating in Morocco. Poult Sci. (2018) 97:4057–62. doi: 10.3382/ps/pey271, PMID: 29982730

[24] Schachner A Gonzalez G Endler L Ito K Hess M. Fowl adenovirus (FAdV) recombination with intertypic crossovers in genomes of FAdV-D and FAdV-E, displaying hybrid serological phenotypes. Viruses. (2019) 11:1094. doi: 10.3390/v11121094, PMID: 31779121 PMC6950264

[25] World Organisation for Animal Health (WOAH). Manual of Diagnostic Tests and Vaccines for Terrestrial Animals. Paris: WOAH (2024).

