# Supplementary figures and images for: Evaluation of clinical, analytical, and genotyping performance of Hex L1 real-time PCR coupled with high-resolution melting curve analysis for fowl adenovirus outbreak investigation in Morocco
Source: Front Vet Sci. 2025 Nov 26;12:1654833. doi: 10.3389/fvets.2025.1654833 (PMC12689382; doi:10.3389/fvets.2025.1654833)

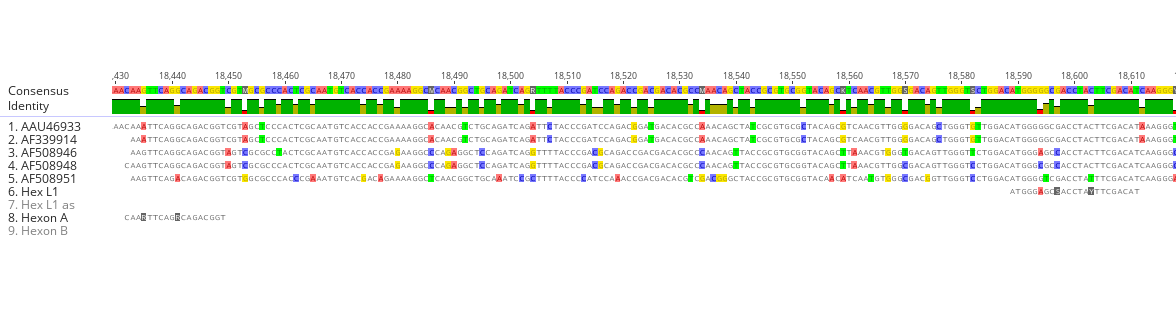

Supplement: SUPPLEMENTARY FIGURE S1 — Multiple sequence alignment of the forward primers (HEXON A and Hex L1) used in this study, shown across five FAdV serotypes corresponding to the five recognized species. [file Image_1.png]

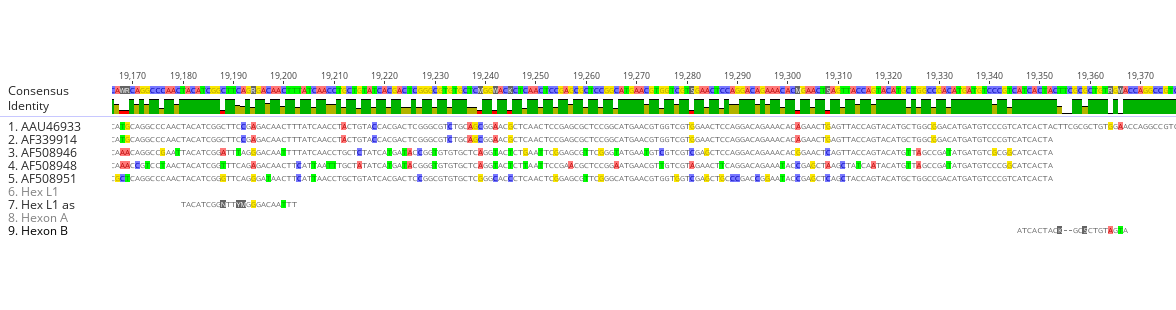

Supplement: Supplementary FIGURE S2 — Multiple sequence alignment of the reverse primers (HEXON B and Hex L1 as) used in this study, shown across five FAdV serotypes corresponding to the five recognized species. [file Image_2.png]
