# Supplementary material for: Evaluation of clinical, analytical, and genotyping performance of Hex L1 real-time PCR coupled with high-resolution melting curve analysis for fowl adenovirus outbreak investigation in Morocco
Source: Front Vet Sci. 2025 Nov 26;12:1654833. doi: 10.3389/fvets.2025.1654833 (PMC12689382; doi:10.3389/fvets.2025.1654833)
Supplement: Supplementary file 4 [file Table_1.docx]

| **Conc.** | **Rep1** | **Rep2** | **Rep3** | **Mean** | **SD** | **CV%** |
| --- | --- | --- | --- | --- | --- | --- |
| 2.2 | 27.30 | 27.23 | 27.19 | 27.24 | 0.054 | 0.20 |
| 5.3 | 22.35 | 22.46 | 22.45 | 22.42 | 0.10 | 0.45 |
| 7.0 | 20.55 | 20.49 | 20.49 | 20.51 | 0.053 | 0.26 |
| 9.1 | 10.64 | 10.52 | 10.58 | 10.58 | 0.074 | 0.70 |

Table 1: Intra-assay (triplicates from one run)

Table 2: Inter-assay (means of triplicates across 3 days)

| **Conc.** | **Day1 mean** | **Day2 mean** | **Day3 mean** | **Overall mean** | **SD** | **CV%** |
| --- | --- | --- | --- | --- | --- | --- |
| 2.2 | 27.15 | 27.60 | 27.58 | 27.44 | 0.29 | 1.06 |
| 5.3 | 22.55 | 22.49 | 22.53 | 22.52 | 0.04 | 0.18 |
| 7.0 | 20.10 | 20.75 | 20.53 | 20.46 | 0.36 | 1.76 |
| 9.1 | 6.40 | 6.66 | 6.55 | 6.54 | 0.11 | 1.75 |
